# The CSF-1-receptor inhibitor, JNJ-40346527 (PRV-6527), reduced inflammatory macrophage recruitment to the intestinal mucosa and suppressed murine T cell mediated colitis

**DOI:** 10.1371/journal.pone.0223918

**Published:** 2019-11-11

**Authors:** Carl L. Manthey, Beverley A. Moore, Yanqing Chen, Matthew J. Loza, Xiang Yao, Hao Liu, Stanley M. Belkowski, Holly Raymond-Parks, Paul J. Dunford, Francisco Leon, Jennifer E. Towne, Scott E. Plevy

**Affiliations:** Janssen Research & Development, LLC, Pennsylvania, United States of America; Duke University, UNITED STATES

## Abstract

**Background & aims:**

Originally believed to be primarily a disorder of T-cell signaling, evidence shows that macrophage-lineage cells also contribute to the pathogenesis of Crohn’s disease (CD). Colony stimulating factor-1 (CSF-1) is a key regulator of the macrophage lineage, but its role in CD has not been well established. We examined transcriptional data from CD mucosa for evidence of CSF-1 pathway activation and tested JNJ-40346527 (PRV-6527), a small molecule inhibitor of CSF-1 receptor kinase (CSF-1R), for its ability to inhibit disease indices in murine colitis.

**Methods:**

A CSF-1 pathway gene set was created from microarray data of human whole blood cultured *ex vivo* with CSF-1 and compared to a TNFα-induced gene set generated from epithelial-lineage cells. Gene set variation analysis was performed using existing Crohn’s mucosa microarray data comparing patients who either responded or failed to respond to anti-TNFα therapy. Commencing day 14 or day 21, mice with T-cell transfer colitis were treated with vehicle or JNJ-40346527 until study termination (day 42). Endpoints included colon weight/length ratios and histopathology scores, and macrophage and T cells were assessed by immunohistochemistry. Mucosal gene expression was investigated using RNAseq.

**Results:**

Both the CSF-1 and the TNFα gene sets were enriched in the colonic mucosal transcriptomes of Crohn’s disease and in mouse colitis, and expression of both gene sets was highest in patients who did not respond to anti-TNFα therapy. In these patients neither set was reduced by therapy. In the mouse model, JNJ-40346527 inhibited the increase in colon weight/length ratio by ∼50%, reduced histological disease scores by ∼60%, and reduced F4/80^+^ mononuclear cell and CD3^+^ lymphocyte numbers. RNAseq analysis confirmed the CSF-1 gene set was sharply reduced in treated mice, as were gene sets enriched in “M1” inflammatory and “M0” resident macrophages and in activated T cells.

**Conclusions:**

CSF-1 biology is activated in Crohn’s disease and in murine T cell transfer colitis. Inhibition of CSF-1R by JNJ-40346527 was associated with attenuated clinical disease scores and reduced inflammatory gene expression in mice. These data provide rationale for testing JNJ-40346527 (PRV-6527) in human inflammatory bowel disease.

## Introduction

Crohn’s disease (CD) is a chronic inflammatory bowel disorder believed to result from loss of tolerance to gut derived commensal bacteria in the context of genetic susceptibility and an environmental trigger. It was generally believed that these factors led to disordered T-cell signaling resulting in the direct and indirect release of high levels of inflammatory mediators such as TNFα. As such, antibodies including infliximab and adalimumab that block TNFα have become an important therapeutic strategy for mitigating disease. However, only 40–50% of patients realize adequate response to this therapy with even fewer achieving full remission, and over time some patient populations will lose response. Thus, additional treatment options are required.

More recently, cells of the macrophage lineage have been implicated in in driving disease pathology. Macrophages are present constituently in all bodily tissues where they mediate multiple processes in both health and disease. In general, these resident “M0” macrophages function as sentinels to defend against pathogens. They are essential for tissue homeostasis, acting as non-inflammatory scavengers of commensal bacteria and senescent cells, assisting in the maintenance of regulatory T cells, and promoting tissue cell renewal by production of growth factors. Resident macrophages are present in particularly high numbers in tissues in contact with the external environment such as the lung and gastrointestinal tract where analysis of their secreted mediator profile indicates that they produce high levels of IL-10 and exhibit an anti-inflammatory phenotype.[1, 2] However, during inflammatory events, macrophage numbers can increase 3- to 4-fold due primarily to recruitment and differentiation from bone marrow derived monocytes. Secreted mediator profiling indicates these infiltrating cells exhibit a pro-inflammatory “M1” phenotype, producing tumor necrosis factor alpha (TNFα), IL-1, IL-6, and IL-23, and are capable of activating T-cells.[3–5] Exemplified by the development of therapeutic anti-TNFα monoclonal antibodies, these cytokines and T cell activation are validated targets in human disease, and targeting the inflammatory pathways associated with the monocyte/macrophage lineage has long been a focus of drug discovery for a variety of disease indications.

Macrophage numbers are increased markedly in the inflamed mucosa of patients with inflammatory bowel disease (IBD), and the ontogeny and functions of various subsets of macrophage-like cells in the bowel have been the topic of numerous investigations and extensively reviewed.[5–8] At a high level, two broad categories immerge, consistent thematically with the bifunctional roles described above. In healthy mucosa, CD68^+^ CD14^lo^ macrophages form a network at the subepithelial lamina propria.[9] When isolated, mucosal macrophages express modest levels of TNFα and high levels of IL-10. In contrast, Crohn’s disease (CD) and ulcerative colitis (UC) are characterized by a marked increase of CD14^+^ cells that have been recently recruited as monocytes, express high levels of TNFα and IL-23, and have potent capacity to activate T cells.[10, 11] Similar data has emerged in mice where mucosal F4/80^hi^ CX3CR1^hi^ macrophages are numerically dominant in healthy animals, and these resident macrophages express high levels of IL-10.[5] While the F4/80^hi^ CX3CR1^hi^ macrophages increase numerically during dextran sulfate sodium (DSS) colitis and the T cell transfer (TCT) model of colitis, they are superseded by a larger increase in F4/80^int^ CX3CR1^int^ cells that have differentiated from recently recruited monocytes.[5, 8] Mirroring the recruited CD14^+^ cells in humans, F4/80^int^ CX3CR1^int^ cells in mice persistently express high levels of TNFα and IL-23 and have potent capacity to activate T cells, indicative of an active role in chronic inflammation.

Although many ligand/receptor interactions regulate macrophage-lineage proliferation and differentiation, among these, the colony stimulating factor 1 receptor (CSF-1R) and its ligands, CSF-1 and interleukin-34, are prominent. In mice, genetic deletion of CSF-1R results in marked to moderate reductions in macrophage-lineage cells in most tissues examined, together with marked reduction in Langerhans cells and partial reductions in some dendritic cell populations.[12] A similar, albeit milder, phenotype occurs in mice with CSF-1-deficiency, while IL-34-deficient mice exhibit selective reductions in Langerhans and microglial cells.[13] Although CSF-1 is expressed at homeostatic levels in healthy tissues, expression by stromal cells can be induced by TNFα and IL-1.[14] Moreover, some T cells may also express CSF-1 upon antigen stimulation.[15] These data are consistent with observations that CSF-1 mRNA was elevated in both Crohn’s and UC mucosa[16] and with the hypothesis that CSF-1R activation may mediate part of the disease related expansion of the macrophage population. Experimental models of colitis also support a role for the CSF-1R pathway, although results from specific models are mixed.[17–20]

JNJ-40346527 (PRV-6527) is a selective small molecule inhibitor of the tyrosine kinase domain of CSF-1R and thereby inhibits CSF-1R signaling. The molecular and cellular pharmacology and safety of JNJ-40346527 in a phase IIA study in rheumatoid arthritis were reported previously.[21] In the current work, we assessed the ability of JNJ-40346527 to suppress CSF-1R-driven biology in the murine T-cell transfer (TCT) model of colitis to determine whether it would impact inflammation and disease indices. To further support a role for CSF-1-R in CD, we generated a CSF-1-inducible gene set. Using gene set variation analysis (GSVA), the gene set was found to be highly enriched in a previously published microarray dataset derived from Crohn’s patient biopsies. GSVA was carried out on murine colonic mucosa to confirm inhibition of the CSF-1 pathway and to examine the impact of treatment on genes enriched in macrophages and T cells.

## Materials & methods

### Reagents

JNJ-40346527, 4-cyano-N-[2-(4,4-dimethylcyclohex-1-en-1-yl)-6-(2,2,6,6-tetramethyl-tetrahydro-2H-pyran-4-yl) pyridin-3-yl]-1H-imidazole-2-carboxamide (abbreviated JNJ527), was prepared at Janssen as described.[22] JNJ-40346527 is currently in Phase 2 clinical development in Crohn’s disease, sponsored by Provention Bio. For oral (PO) dose administration at 10 ml/kg, JNJ-40346527 was prepared fresh daily in vehicle (0.5% (w/v) METHOCEL*F4M Premium hydroxypropyl methylcellulose in deionized water).

CNTO5048 is an IgG2a anti-mouse TNFα monoclonal antibody that binds soluble and transmembrane TNFα and prevents interaction of TNFα with its receptor. CNTO6601, an IgG2ak anti-tumor associated epithelial membrane protein (EMP) monoclonal antibody was used as an isotype control. Both were designed and generated at Janssen R&D (Spring House, PA). The antibodies were formulated at 0.3 mg/ml in sterile phosphate buffered saline and dosed at 10 ml/kg (3 mg/kg) by intraperitoneal (IP) injection.

### Generation of human CSF-1 and TNFα-induced gene sets

To identify CSF-1 induced genes, whole blood from three healthy donors was drawn into EDTA vacutainer tubes. Participant blood samples acquired for generation of the CSF-1 gene signature were obtained subsequent to IRB approval from an independent ethics review board, Quorum Review IRB, Cambridge, MA, according to the principles of the Declaration of Helsinki. All subjects provided written informed consent.

Two aliquots of 1 ml of blood were transferred to sterile 2 ml Eppendorf tubes. Recombinant human CSF-1 (PeproTech) was added to one of the two replicate tubes to a final concentration of 50 ng/ml, with the second replicate tube retained as an unstimulated control sample. Tubes were affixed to a tube rotator and incubated at 37°C, 5% CO_2_. Aliquots (100 μL) were collected from all tubes at 4 and 24 hr, and RNA isolated using a MagMAX total RNA isolation kit (ThermoFisher Scientific) according to the manufacture’s protocol. cDNA was generated and aliquoted onto GeneChip Human Transcriptome Array 2.0 (ThermoFisher) and analyzed via the manufacturer’s online proprietary software. The CSF-1 gene set was comprised of 57 genes (ARHGAP18, ARHGAP22, ATF3, BCAT1, CASP1, CCL8, CCR2, CD14, CD163, CLEC5A, COL7A1, CSF1R, CTSB, DSE, EPB41L3, FBP1, FCN1, FCRLA, FLJ42418, FMNL3, FPR3, FUCA1, HIVEP3, HLA-DPA1, HLA-DRA, HNMT, IFNB1, IQSEC2, KYNU, LAP3, LRP1, MAFB, MARCH1, MARCKSL1, MERTK, MRAS, MS4A14, MS4A6A, MS4A7, MYOF, NAPSB, OAS1, OLR1, RIN2, SCD, SDS, SEMA6B, SIGLEC1, SIGLEC16, STARD4, TFEC, TGM2, THBS1, TMEM180, VCAN, VSIG4, ZNF330) with geometric means >2-fold relative to unstimulated control conditions (p≤0.05) at either 4 or 24 hrs. The microarray data is available in GEO DataSets under accession no. GSE136440.

To create a TNFα-induced gene set, triplicate cultures of A549, HT-29, HEK293, and primary human keratinocytes were cultured without (unstimulated control) or with 10 ng/ml human recombinant TNFα (PeproTech) for 4-to-24-h and gene expression analyzed by Affymetrix GeneChip® HG-U133+2 array or HG-U133+PM array plate. TNFα-induced gene set was comprised of 25 genes (ATXN1, BID, BIRC3, C3, CCL20, CD83, CLDN1, CXCL1, CXCL2, CXCL5, CYLD, DRAM1, IFNGR1, IKBKE, IL32, IL8, KIF3C, KLK10, LAMC2, NFKB2, NFKBIA, OPTN, SOD2, TNFAIP3, TNIP1) with geometric means ≥1.5-fold relative to unstimulated control conditions (p<0.05) in at least two cell types. The microarray data is available in GEO DataSets under accession no GSE136439.

### Gene set variation analysis (GSVA) of human Crohn’s mucosa

Transcriptomic profiles of individual RNA samples from Crohn’s disease and healthy control mucosal biopsies have been previously published (NCBI/NIH GEO DataSet GSE16879). [23] Raw cel files from Affymetrix GeneChip® HG-U133+2 arrays were subjected to robust multi-array average normalization, and paired analysis was conducted for before and after treatment samples from the same patient for differences in gene expression due to drug treatment. GLM (general linear model) was used to obtain gene expression differences between diseased and healthy controls. GSVA was performed as described by Hänzelmann,[24] mapping the CSF-1 and TNFα-induced gene sets onto the GSE16879 Crohn’s disease and healthy control normalized dataset. In addition, gene lists enriched for human M0 and M1 macrophages were applied to the GSE16879 data set, and GSVA was performed. The M1 and M0 gene lists were derived from a matrix of genes reported to have low/limited expression outside the hematopoietic system but to have high differential expression in subsets of lymphoid and myeloid cells (see S2 Fig).[25]

### T cell transfer (TCT) model of colitis

The in-life portions of the murine colitis model studies were performed by Bolder BioPATH, Boulder Colorado. Female Balb/C mice (12 weeks of age) and female Fox Chase C.B-17 SCID mice (5–6 weeks of age) were obtained from Harlan, Inc., Indianapolis, Indiana. Animals were maintained under a 12 hr light/dark cycle and were provided with standard rodent chow and water *ad libitum*. Mice were acclimated to animal facility housing for a minimum of 5 days prior to initiation of the study. All animal handling and housing procedures were approved and conducted in accordance with Bolder BioPath Institutional Animal Care and Use Committee guidelines [IACUC Protocol #BP12-100].

**Fig 1:**
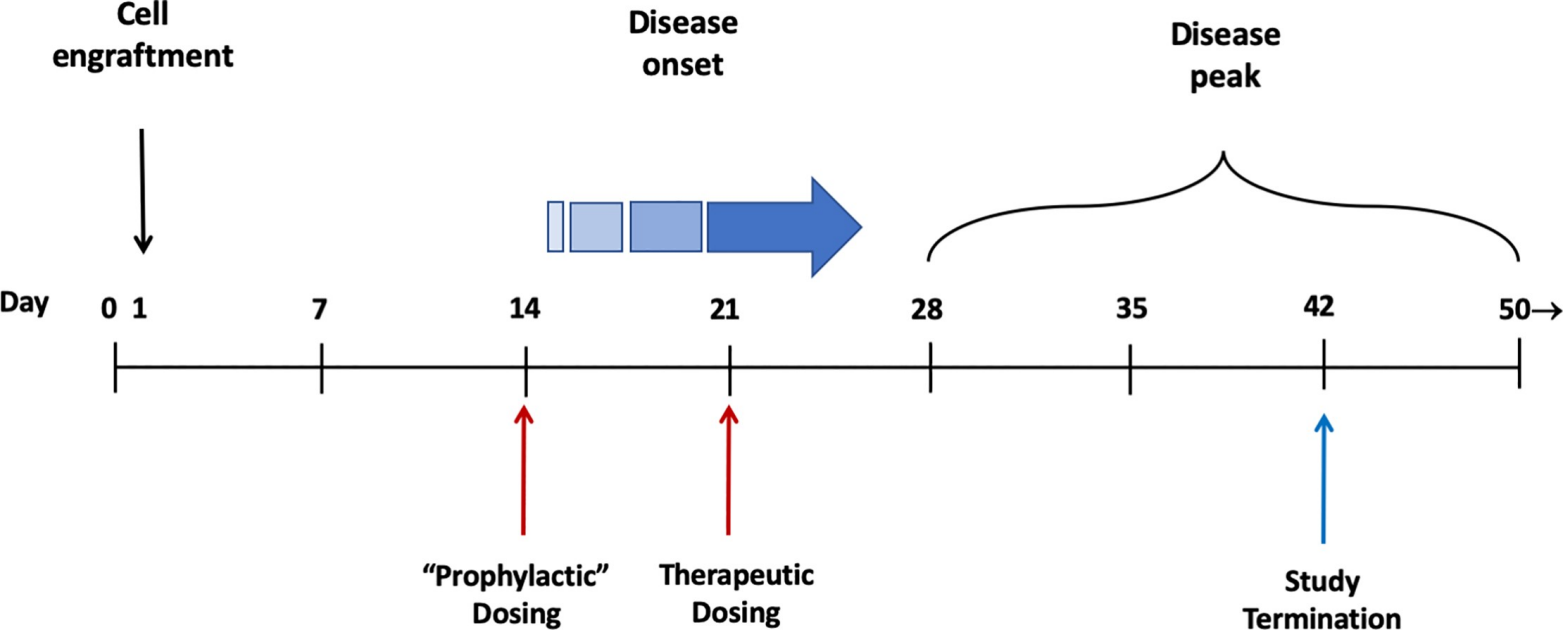
Schematic outlining T-cell colitis disease progression time course.

Colitis was induced as described previously.[26] Single cell suspensions were prepared from the spleens of BALBc mice by trituration and passed through 40 μm cell separator mesh (BD Falcon). Red cells were lysed with RBC lysis buffer and T cells were collected using a MACS CD4^+^ separator kit (Miltenyi Biotech). Naïve T cells were then isolated by FACS sorting using a specific antibody against CD45RB (Dynal® Mouse CD4 Negative Isolation kits, Invitrogen, CA). Female Fox Chase C.B-17 SCID, which lack B and T cells, were inoculated intraperitoneally (IP) with a minimum of 4×10^5^ CD4^+^CD45RB^high^ naïve T cells. Animals so treated develop progressive and unrelenting inflammatory colitis. Two dosing paradigms were employed to determine the efficacy of JNJ-40346527: “Prophylactic” dosing was initiated on day 14, a time when naïve T cells are expected to have fully engrafted, but before clinical signs of disease (e.g., weight loss) are manifest (Fig 1). “Therapeutic” dosing commenced on day 21 when mice exhibit clinical signs of disease as noted in the following schematic. Treatment groups are summarized below and in greater detail in S1 Table.

### Study 1—Dose response, prophylactic dosing

On study day 0, SCID recipient mice were inoculated with naïve T cells as noted above. On study day 14, mice were randomized by body weight into treatment groups receiving vehicle, or 5, 10, or 20 mg/kg JNJ-40346527 administered orally (PO) daily (QID at 24 hr intervals) through day 41. SCID mice without T cell transfer (naïve) were included as disease-free controls.

### Study 2—Prophylactic vs. therapeutic dosing

SCID mice underwent T cell transfer on day 0. On study day 14, all mice were randomized by body weight into treatment groups, and prophylactic treatment was initiated with 15 mg/kg JNJ-40346527 or vehicle. A third group of mice received vehicle until day 21 at which time therapeutic dosing of compound was initiated. Dosing was administered PO, QID through day 41. As a positive control, other groups of mice were treated intraperitoneally (IP), every third day (Q3D) commencing day 21 with either PBS, or with 3 mg/kg CNTO5048 (anti-mouse TNFα),or 3 mg/kg isotype control. Study 2 included additional parallel groups of mice designated for RNAseq analysis, treated under therapeutic dosing conditions. Specifically, additional SCID mice with adoptive T cell transfer were dosed PO, QD with vehicle or 15 mg/kg JNJ40346527, or IP, Q3D with PBS, 3 mg/kg CNTO5048 or 3 mg/kg isotype control. Also included for RNAseq analysis were naïve SCID mice (no T cell transfer) and a group of immunocompetent Balb/c mice.

### Clinical and histological assessment of TCT colitis

All mice were weighed on study day 0 and then every other day commencing on day 14. Final body weights were determined on day 41 with subsequent tissue harvest on day 42 following an overnight fast. Percent change in body weight relative to day 14 (Study 1) or day 0 (Study 2) was reported. At necropsy, following an overnight fast, the entire colon was harvested from each animal, inspected visually, measured for length, and weighed. Results were reported as weight/length ratio. Colons were fixed in 10% neutral buffered formalin (NBF) for 48 hours, transferred to 70% ethanol and embedded in paraffin within 1 week of fixation. Two paraffin blocks were prepared from each animal: 1 block containing 3 pieces of proximal colon, and 1 block containing 3 pieces of distal colon (~1 cm long each). Paraffin embedded blocks were sectioned, processed for H&E and scored for pathological changes by a trained pathologist (Bolder BioPATH). Four parameters (i.e., inflammation, glandular loss, erosion, and hyperplasia) were scored and summed to arrive at a sum of histopathology scores. Neutrophil infiltrate fraction was estimated separately. Details of the scoring method and the method to estimate neutrophil infiltrate fraction are provided in S1 Text.

Immunohistochemical staining for F4/80 and CD3 was performed at HistoTox Labs, Boulder, CO. Detection of staining was accomplished by capturing digital images from three tissue sections at 40X using Aperio ScanScope XT (Leica Biosystems, Danvers, MA). Staining was quantified using Aperio GENIE® pattern recognition software which detects the number and intensity of positively stained pixels and the total tissue area. Results were reported as total positive pixels normalized to tissue area.

### Analyses of gene expression in TCT colitis

On study day 42 mice were euthanized and mucosa from the middle 2/3 of the colon was harvested by separating the mucosa from the muscle layer as previously described.[27] Tissues were collected in Matrix Lysis-D tubes, immediately snap frozen in liquid nitrogen and stored at -80 C for subsequent RNA extraction. Mucosal homogenates were prepared in 1 ml RLT buffer with the aid of a Bead Ruptor bead mill (Omni International) and total RNA was isolated using a Qiagen RNeasy Mini kit according to manufacturer’s protocol. RNAseq was performed by BGI Americas Corporation (Cambridge, MA). FASTQ files were obtained from BGI and processed using ArrayStudio (OmicSoft/QIAGene, Research Triangle Park, NC). QC passed FASTQ files were mapped to the mouse mm10 reference genome using gene model ENSEMBLE.R72 to obtain gene expression quantifications. Deseq2 and GLM (General Linear Model) methods implemented in ArrayStudio were used to compare gene expression differences between treatment groups using the original RNA sequence read count data at individual gene level. The RNAseq source data may be found at NCBI BioProject accession number PRJNA563096.

GSVA was performed using the CSF-1 and TNFα-induced gene lists (after translation to mouse homologues) and gene lists enriched for M0 and M1 macrophages and activated Th1 T cells. The cell-type lists were derived from a matrix of genes reported to have low/limited expression outside the hematopoietic system but to have high differential expression in 24 subsets of lymphoid and myeloid cells.[28] For use herein, gene sets were derived by sorting the top 30 genes by signal intensity for the indicated cell type (lists are provided in S2 Table, S3 Table, and S4 Table). Overlap in the gene sets was nominal. Ingenuity® Pathway Analysis (IPA®), a web-based software application, was used to assess enrichment of gene sets in canonical pathways and biological processes.

### Statistical analyses

Statistical analyses of clinical indices were performed using Robust Analysis of Variance (ANOVA). The analysis included homogeneity of variance testing (Levine’s test) for assessing whether ANOVA should be performed on transformed values to stabilize variance. Following variance assessment, data was robust transformed to adjust for extreme and outlying values using the Huber-Bickel-Tukey method (a form of M-estimation). The robust transformed data was then fitted by ANOVA and Dunnett’s multiple comparison post hoc testing to evaluate significance of mean differences between select treatments. GSVA scores were analyzed by one-way ANOVA followed by the two-stage linear step-up procedure of Benjamin, Krieger and Yekutieli to determine false discovery rate (FDR) with cutoff at p≤0.05. Discrete data or data consisting of score counts was analyzed by Kruskal-Wallis and Dunn’s multiple comparison test, both nonparametric alternatives to ANOVA.

## Results

### Gene set variation analysis (GSVA) supports a role for CSF-1 in Crohn’s disease

To correlate CSF-1R-driven biology in disease datasets, a CSF-1 gene set was derived by identifying fifty-seven genes induced >2-fold by CSF-1 in ex vivo whole human blood cultures (see Methods for full list). Blood monocytes are an exemplary target of CSF-1, and consistent with the role of CSF-1 as a monocyte/macrophage regulatory factor, Ingenuity Pathway Analysis identified “activation of phagocytes” as the top enriched disease and function category (p = 4x10^-10^).

**Fig 2:**
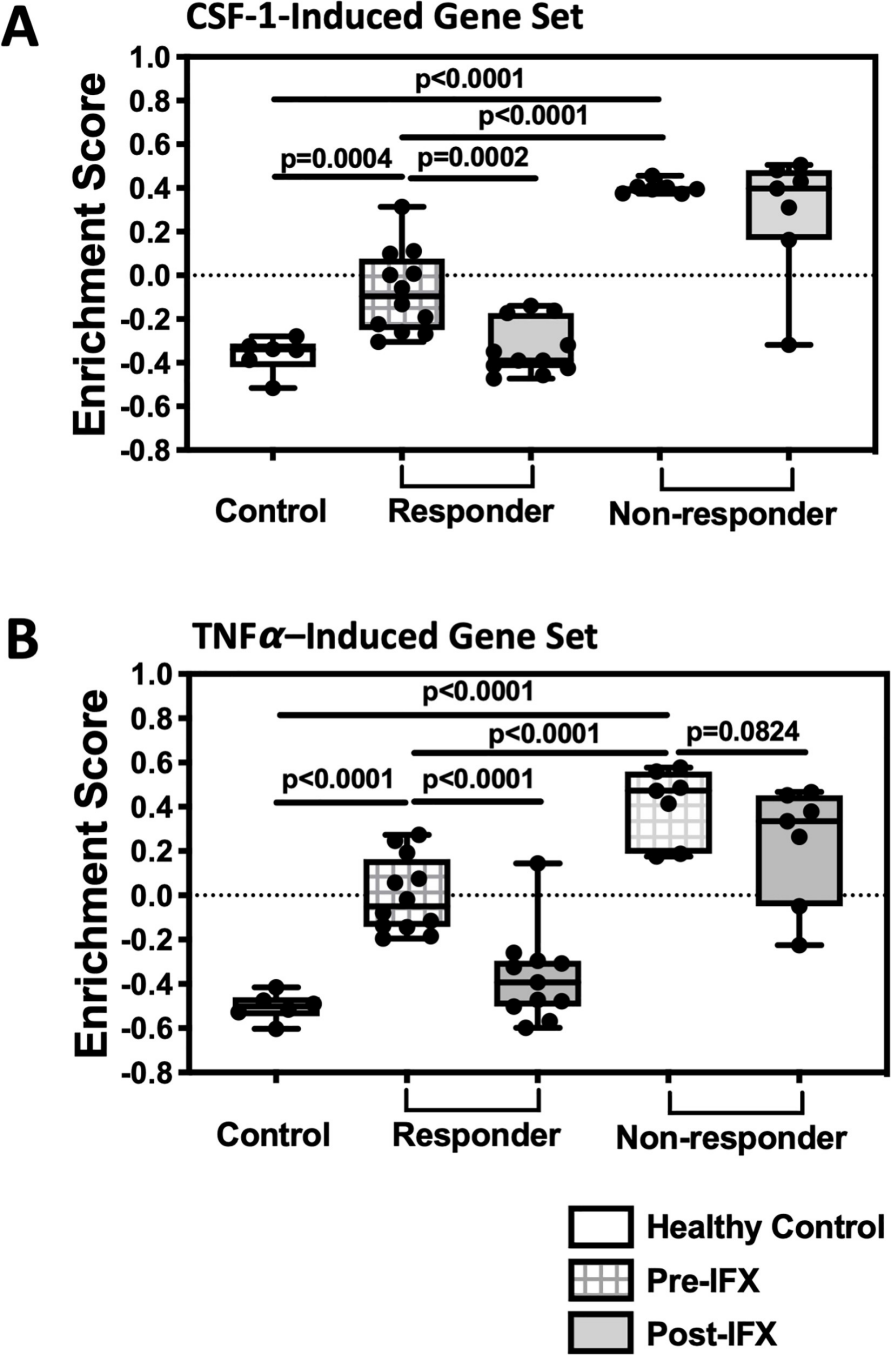
Gene set variation analysis (GSVA) of CD colonic biopsies indicates enriched CSF-1-mediated biology. A literature-derived colon mucosa biopsy microarray dataset (GSE16879) was submitted to GSVA using a CSF-1-induced gene set (A) and a TNFα-induced gene set (B), and enrichment scores (ES) were illustrated using five quartile box and whisker plots (95% confidence interval). The colon mucosal biopsies were from healthy controls (clear boxes) or from subjects with CD before (cross-hatched boxes) and after (grey boxes) infliximab therapy.

A transcriptomic analysis of CD colonic mucosal biopsies before and after patient treatment with infliximab (anti-TNFα antibody) was published previously.[23] Using this CD dataset and the CSF-1 and TNFα gene sets, GSVA was performed in CD colonic tissue. Patients were resolved into those with good response to infliximab vs. non-responding patients. Response was assessed by endoscopy and histology in CD patients 4 to 6 weeks after the first infliximab treatment and responders were defined as having complete mucosal healing. When compared to the healthy control group, the CSF-1 gene set enrichment scores were robustly elevated prior to infliximab treatment in CD patients who did not meet the response criteria (Fig 2). Enrichment of the CSF-1-induced gene set was not reduced following infliximab treatment, consistent with non-response. The CSF-1 pathway signal was also significantly increased at baseline in patients who subsequently met the response criteria, although to a lesser extent than observed in non-responders. This signal was lowered to the level of healthy mucosa following clinically effective treatment. Further, the expression of CSF-1R mRNA was consistent with increased CSF-1R pathway activity, viz., at baseline relative to healthy controls, expression of CSF-1R was significantly 1.92-fold higher in responders and 2.78-fold in non-responders and was restored to healthy levels with treatment only in responders. S5 Table provides a summary of the CSF-1 gene set in this Crohn’s cohort at the individual gene level. Collectively, the data supported a hypothesis that CSF-1 driven biology is elevated in Crohn’s disease mucosa, even more so in infliximab non-responders.

TNFα released from immune cells acts on a wide variety of cell types including epithelial cells during mucosal inflammation. GSVA was similarly performed using a TNFα-induced gene set consisting of twenty-five genes significantly up-regulated by TNFα in multiple epithelial cell lines (Fig 2A. See Methods for full list). Similar to the CSF-1 gene set, the TNFα gene set was enriched in Crohn’s mucosa, was highest in infliximab non-responders, and restored toward healthy levels only in the responders (Fig 2B). The CSF-1 and the TNFα gene sets did not intersect at the gene level, i.e., there were no common genes.

### JNJ-40346527 reduced severity of murine colitis when dosed prophylactically or therapeutically

**Fig 3:**
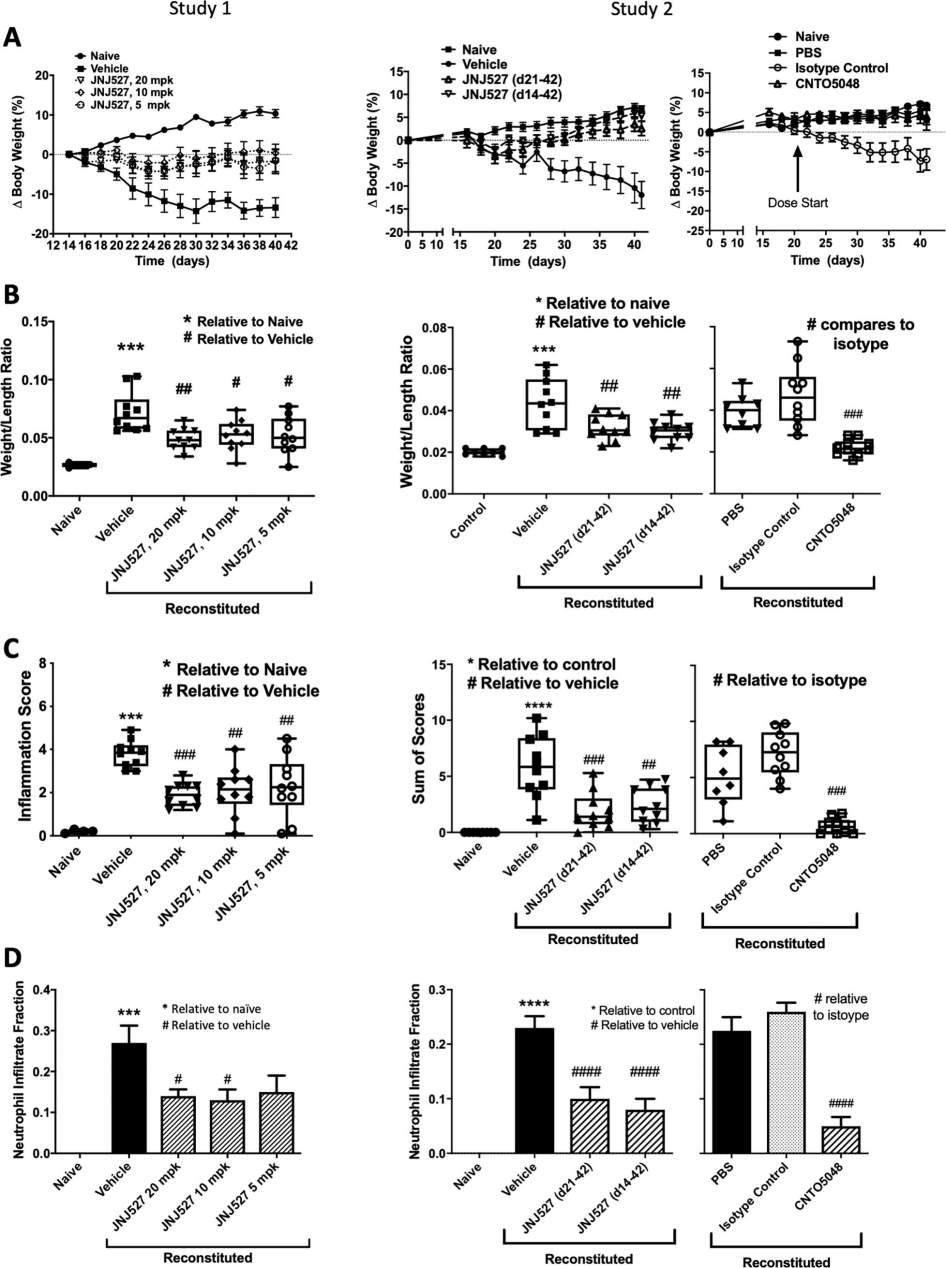
Clinical and histopathology scores for healthy control mice and in T cell reconstituted colitic mice in studies comparing JNJ-40346527 to vehicle. CNTO5048 (anti-TNFα) served as a positive control. (A) Change in body weight; (B) colon weight/length (W/L) ratio; (C) Sum of histological scores; (D) Estimated fraction of cellular infiltrate that were neutrophils. Naïve control group, n = 4 (Study #1) or n = 8 (Study #2). PBS group, n = 8. All remaining groups, n = 10. Data are mean ± SEM (A and D) or five quartile box plots, 95% confidence interval (B and C). *^#^P<0.05 relative to naïve or vehicle control, respectively. #, p<0.05; ##, p<0.01; *** or ###, p<0.001; **** or ####, p<0.0001.

Transfer of naive T cells into the severe combined immunodeficiency (SCID) mice provides a highly reproducible and easily manipulated model of colitis that has characteristics similar to IBD in humans.[26] To assess the net role of CSF-1 in experimental colitis we employed the TCT-colitis model together with a selective inhibitor of CSF-1R kinase, JNJ-40346527. In each of two studies weight losses averaging 10–15% occurred by day 40 in the oral vehicle-treated or isotype control-treated disease groups while the naïve, disease-free group experienced 5–10% weight gain (Fig 3A). Treatment with JNJ-40346527 significantly prevented disease-induced body weight loss at all three dose levels in Study 1. In Study 2, body weight gain was restored by both prophylactic and therapeutic dosing. Study 2 included a positive control group treated with anti-TNFα and the restoration of weight gain by JNJ-40346527 was similar to that achieved with anti-TNFα. Unexpectedly, the PBS-treated group in Study 2 did not show weight loss, although subsequent indices of disease were evident. This may have been the result of the smaller group size (See S1 Table) and chance accumulation of mice with mild disease. Data provided in S1 Fig shows combined results from two additional studies where PBS treated animals exhibited weight loss similar to vehicle control.

Fig 3B shows that the disease-associated increase in colon weight/length ratio in vehicle-treated mice was significantly reduced by treatment with JNJ-40346527. The therapeutic dosing regimen was found to be equally effective in reducing disease parameters. Histological assessment of disease is shown in Fig 3C as the sum of four scoring parameters, i.e., inflammation, glandular loss, hyperplasia, and erosions. Sum of scores was markedly increased in animals with disease and significantly reduced by treatment with JNJ-40346527 (results for individual components are provided in S6 Table). Further, JNJ-40346527 significantly reduced the disease associated neutrophil infiltrate (Fig 3D). Overall, JNJ-40346527 showed minimal dose-dependency with the numerically maximal effect at 20 mg/kg.

### JNJ-40346527 reduced recruitment of F4/80^+^ macrophages and CD3^+^ T cells during TCT-colitis

**Fig 4:**
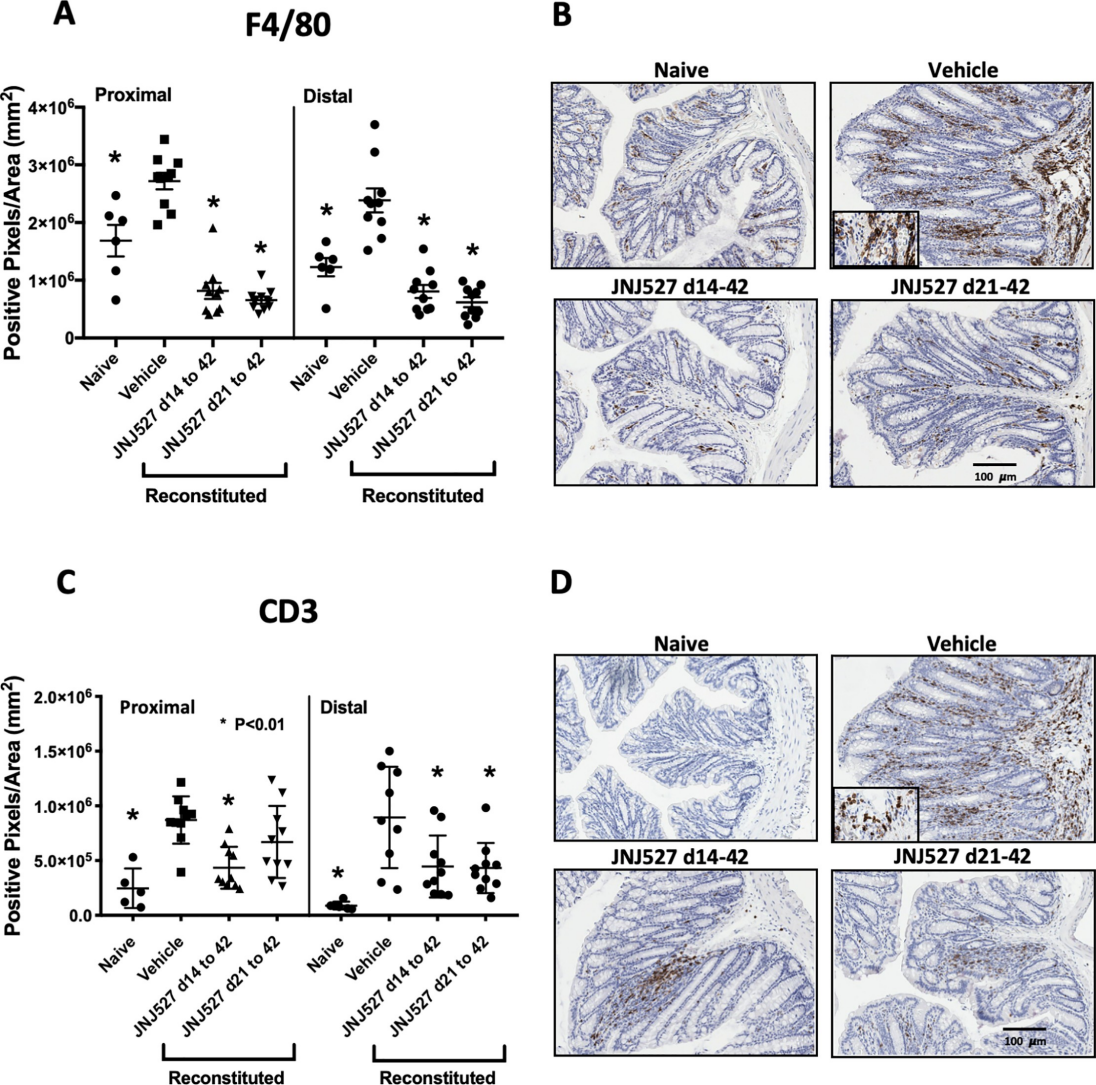
**Colonic mucosa from T cell reconstituted colitic mice stained for F4/80 (A) and (B), and CD3 (C) and (D) antigens**. Data points are positive pixels per mm^2^ determined by digital imaging of three sections per mouse per colonic region. Data are group mean ± SEM shown together with values from each mouse. Naïve control group, n = 6. All remaining groups, n = 10. *P<0.01 relative to respective vehicle control for either proximal or distal colon. Images are representative of sections with median pixel density. Insets represent enlarged images of cellular infiltrates to demonstrate cellular morphology.

We hypothesized that JNJ-40346527 has a direct impact on macrophage activity/phenotype with consequent impact on T cells. Numbers of mucosal F4/80^+^ macrophages and CD3^+^ T cells were investigated by immunohistochemistry in Study 2. F4/80^+^ macrophages were significantly increased in the proximal and distal colon of mice with TCT-colitis compared to naïve SCID mice (Fig 4A and 4B). Staining was principally in the lamina propria. JNJ-40346527, administered prophylactically or therapeutically, blocked completely the disease-related increase F4/80^+^ cells. CD3^+^ staining was, as expected, low/absent in the naïve control SCID mice but CD3^+^ cells were abundant in vehicle-treated mice with TCT-colitis (Fig 4C and 4D). The T cell infiltrate was significantly reduced in animals treated with JNJ-40346527.

### Transcriptomic analysis supports CSF-1-driven biology in TCT colitis that is inhibited by JNJ-40346527

To further probe the therapeutic mechanisms of JNJ-40346527, Study #2 included additional parallel cohorts of mice designated for RNAseq of colonic mucosa. TCT colitis was associated with >2000 genes differentially expressed >2-fold when compared to naïve mice. The number of genes reaching significance was reduced 94% in the group treated with JNJ-40346527, an effect similar to CNTO5048. Conversely, when compared to vehicle-treated mice with disease, JNJ-40346527 and CNTO5048 significantly impacted 1091 and 1280 genes, respectively. An overall summary of numbers of genes impacted by disease and treatments is provided in S7 Table.

**Fig 5:**
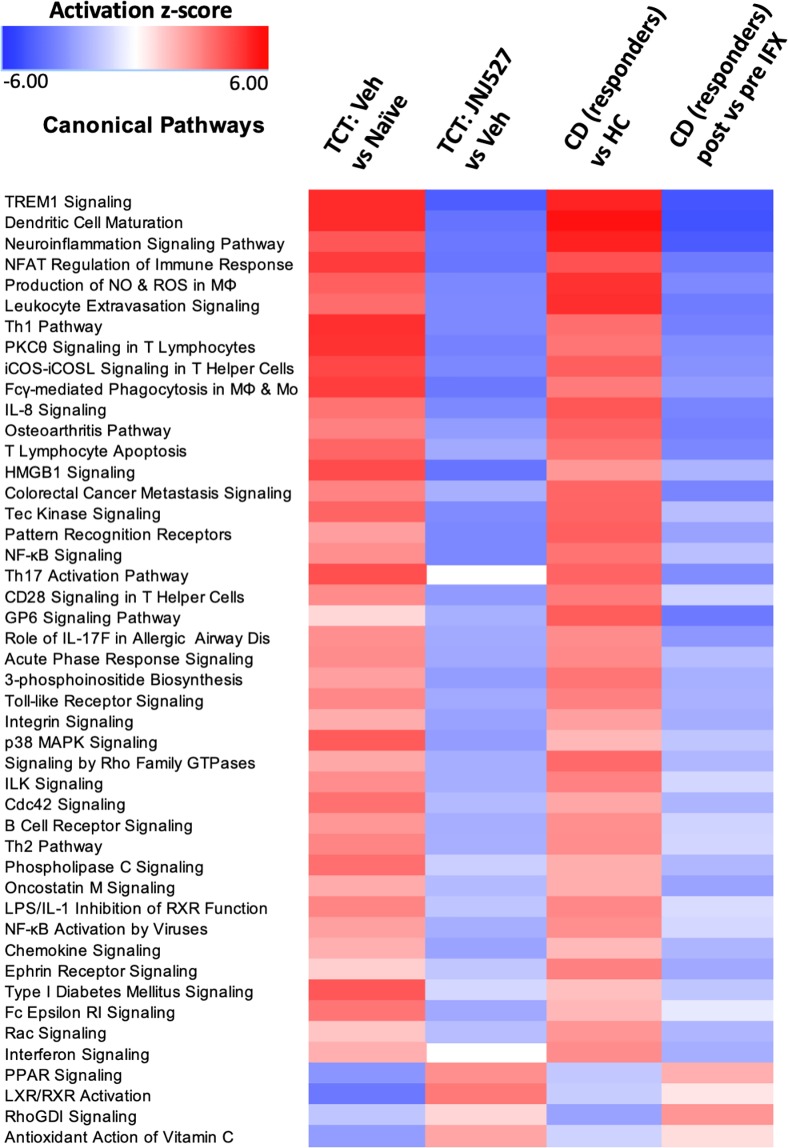
IPA pathway analysis of CD and T cell reconstituted colitic mouse datasets. Data in column 3 represents those patients who responded to infliximab. Differentially expressed genes from the comparisons indicated were used to calculate Z score enrichment in gene sets defining canonical pathways by IPA (2018). The Z scores were used to generate a heat map. Red = enhancement; Blue = suppression. The criteria for inclusion were P ≤ 0.01 and Z>2.7 in any one of the four comparisons. The analysis of all available pathways is provided in S8 Table.

To assess potential translatability of the TCT colitis model to human disease, IPA pathway analysis was performed using the genes differentially expressed in the aforementioned CD dataset (GSE16879) and in the mouse dataset described herein. At the pathway level, there was strong concordance between the mouse model and human disease (Fig 5). Furthermore, treatment of mice with JNJ-40346527 effectively and broadly countered disease pathway activation.

**Fig 6:**
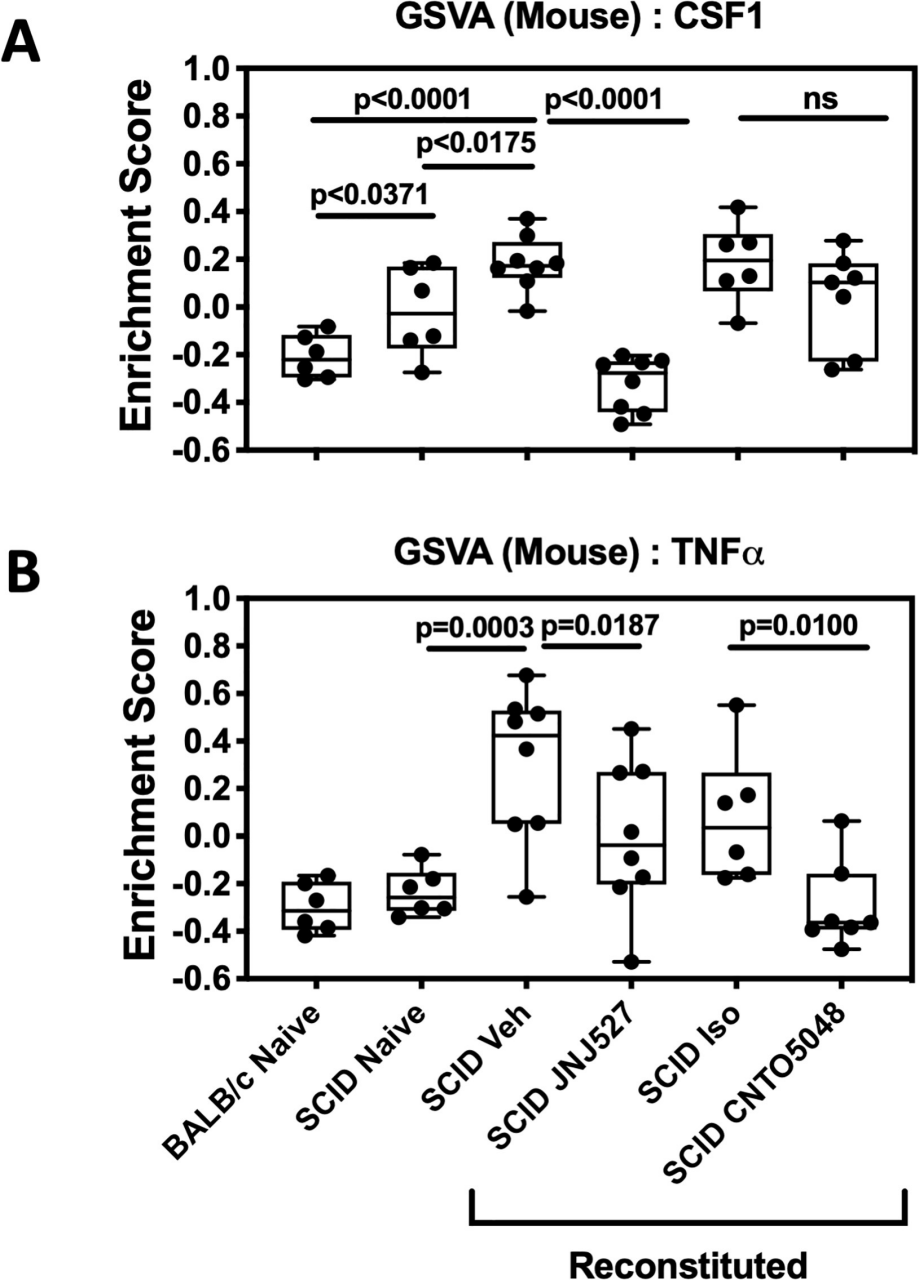
**Gene set variation analyses (GSVA) of mucosal transcriptome from T cell reconstituted colitic mice using murine homologues of the human CSF-1 (A) and TNF**α **(B) induced gene sets**. Data points representing individual mice are plotted in five quartile box plots (95% confidence interval) and demonstrate the increased expression of both gene sets in mouse colon and response to treatment. Naïve and isotype controls, n = 6. All remaining groups, n = 8. Veh = vehicle for JNJ527; Iso = isotype control for CNTO5048.

Next, GSVA was employed to assess CSF-1 pathway strength in the model (Fig 6). Mild elevation in CSF-1 pathway strength was apparent in naïve SCID mice compared to Balb/c, suggesting a compensatory increase in innate pathways in this T and B cell deficient strain. However, similar to CD, expression of the CSF-1 gene set was further enriched in mice with TCT-colitis, and good concordance at the individual gene level existed between Crohn’s disease and the murine model (S5 Table). The CSF-1 gene set also provided an opportunity to assess directly the pharmacodynamics of JNJ-40346527 in disease tissue. Consistent with its mechanism as a CSF-1R inhibitor, JNJ-40346527 caused a sharp reduction in CSF-1-pathway strength to a level that was similar to that in Balb/c mice. In contrast, CNTO5048 had a weak suppressive net effect on the CSF-1 gene set that did not reach significance, although several individual genes were suppressed following JNJ-40346527 treatment (S5 Table). Conversely, CNTO5048 had a marked suppressive impact on the TNFα-induced gene set which was otherwise sharply elevated in the vehicle-treated group, while JNJ-40346527 had a moderate suppressive net effect on the TNFα gene set.

**Fig 7:**
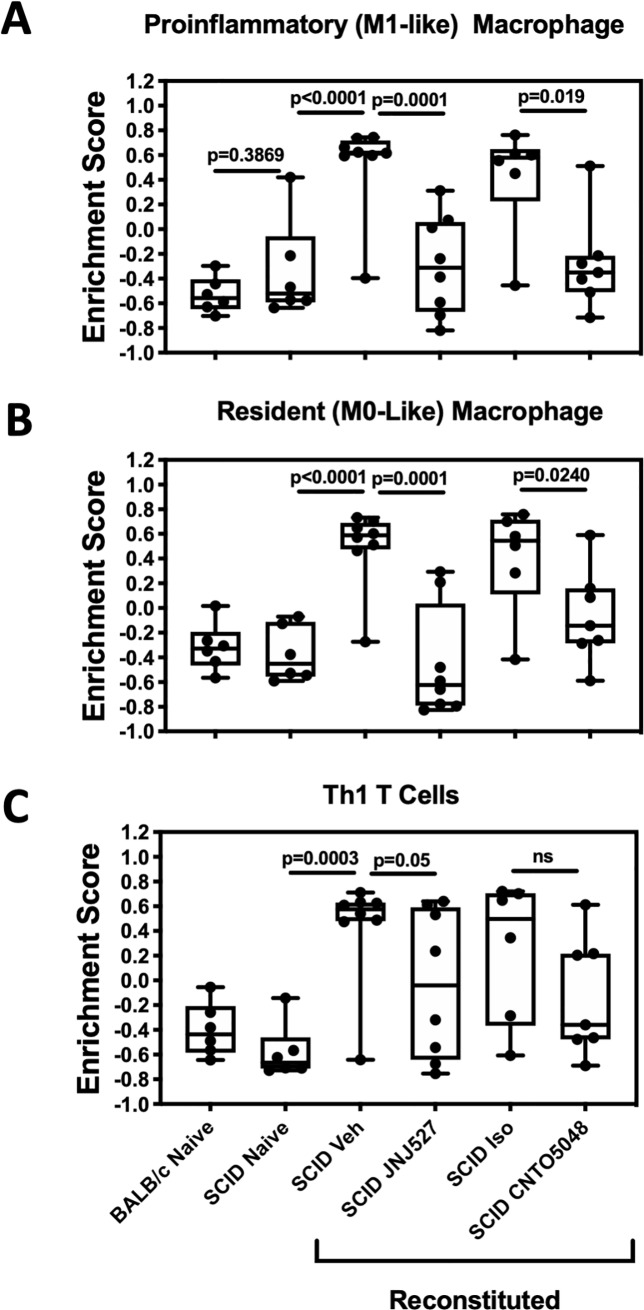
**Gene set variation analyses (GSVA) of mucosal transcriptome from T cell reconstituted colitic mice using gene sets enriched in (A) M1 macrophages, (B) M0 resident macrophages, and (C) activated Th1 T cells**. Data points representing individual mice are plotted in five quartile box plots (95% confidence interval) Naïve and isotype controls, n = 6. All remaining groups, n = 8. Veh = vehicle for JNJ527; Iso = isotype control for CNTO5048.

Although the loss of F4/80^+^ cells suggested a reduction in macrophages, it may also reflect reduced expression of the F4/80 antigen as CSF-1 is reported to induce F4/80 protein expression.[29] To examine further the impact of JNJ-40346527 on the phenotypic composition of mucosal macrophages and to confirm the impact on T cell activation contributing to colitis, we employed further gene set analyses. We utilized a matrix of genes reported to have low/limited expression outside the hematopoietic system but to have high and differential expression within subsets of lymphoid and myeloid cells.[28] From this gene matrix, gene sets were derived enriched in inflammatory “M1” macrophages, resident-like “M0” macrophages and activated Th1 lymphocytes. Consistent with F4/80 immunohistochemistry, GSVA indicated the M1 and M0 macrophage gene sets were enriched in the inflamed mucosa of vehicle-treated mice, while JNJ-40346527 treatment was associated with sharp reductions in both gene sets (Fig 7A and 7B). Similarly, genes enriched in activated Th1 lymphocytes were markedly increased in the inflamed mucosa, and significantly reduced with JNJ-40346527 treatment, consistent with the overall anti-inflammatory activity of the compound in the model (Fig 7C). The full gene sets used herein and expression data at the individual gene level are provided in S2 Table, S3 Table and S4 Table. Data on additional genes that encode cytokines and surface and transcription factor markers used to identify M1 and M0 macrophages, monocytes, and Th1 lymphocytes are provided in Table 1, including chemokines mediating monocyte recruitment. Expression of these genes was increased in TCT colitis and reduced following treatment. Collectively these data further support the conclusion that JNJ-40346527 treatment reduced the recruitment of these cells in TCT colitis.

**Table 1:** Regulation of macrophage, monocyte, T cell-related genes in by TCT colitis and JNJ-40346527.

		TCT vs Naive		JNJ-40346527 vs Vehicle	
Association	Gene Name	Fold Change*	p-value (FDR)	Fold Change*	p-value (FDR)
**M1 macrophage**	Tnf	5.22	<0.0001	-2.31	0.044
	Il1b	7.82	<0.0001	-5.81	<0.0001
	Nos2	4.57	0.0001	-2.99	0.025
**M0 macrophage**	Cd68	2.67	<0.0001	-3.32	<0.0001
	Emr1 (F4/80)	1.46	0.106	-5.47	<0.0001
**Monocyte**	Ccr2	4.23	<0.0001	-3.50	<0.0001
	Ly6c1	4.44	<0.0001	-3.00	0.0018
	Ccl2	6.39	<0.0001	-3.50	0.0018
	Ccl7	10.1	<0.0001	-7.57	<0.0001
**Th1 lymphocyte**	Cd4	3.22	<0.0001	-2.31	0.015
	Tbx21	3.94	<0.0001	-2.29	0.017
	Ifng	3.70	0.0017	-2.72	0.055

*Fold change determined by RNAseq of between expression of SCID mice with TCT colitis (vehicle-treated) and naïve SCID mice or SCID mice with TCT colitis treated with JNJ-40346527 in study 2 vs vehicle treated.

A similar approach was used to examine enrichment of human M1 and M0 macrophage genes in the aforementioned CD colon mucosa dataset. M1 and M0 gene signatures were elevated in CD patients and was elevated greatest in patients who did not respond to infliximab (S2 Fig). Treatment with infliximab restored the signatures to baseline in responders, but had no effect in non-responders, lending support to the hypothesis that a macrophage-mediated disease component is enhanced in patients who do not respond to anti-TNFα therapy.

## Discussion

In this manuscript we report the enrichment of CSF-1-inducible genes in CD colonic mucosa. Enrichment in CD was mirrored by enrichment of CSF-1-inducible genes in murine TCT colitis, wherein gene sets for M1 and M0 macrophages were also enriched. In TCT colitis, the CSF-1R inhibitor, JNJ-40346527, effectively restored the CSF-1 and macrophage gene sets to baseline. Reduction in CSF-1 and macrophage signal strength correlated with diminished disease activity and histological parameters, and in reduced enrichment of a Th1 T-cell associated gene set and reduced CD3^+^ T cells staining. IPA analysis of the TCT-colitis and CD gene signatures indicated good concordance at the level of biological pathways. JNJ-40346527 restored those pathways in TCT colitis toward normal similar to pathway restoration in CD patients responding to anti-TNFα therapy. Overall, the results support the ongoing clinical and translational evaluation of JNJ-40346527 (PRV-6527) in CD.

We demonstrated the utility of a novel gene set induced by CSF-1 in human blood. While the gene set includes genes not previously known to be regulated by CSF-1, IPA functional curation confirmed it to be highly enriched for genes significant to macrophage biology. Robust suppression of the gene set in mice treated with CSF-1R inhibitor further supported its use as an indicator of CSF-1R mediated biology. Using the gene set with GSVA provided strong, contextual evidence of CSF-1R-mediated biology in CD, consistent with prior data indicating increased expression of CSF-1R and its ligands in CD.[16, 30] There was striking similarity between CD and TCT-colitis (see data columns 1, 2, and 4 of S5 Table) when the gene set was viewed at the individual gene level. Taken together, the data highlight the translational value of the gene set. Although blood-derived, the gene set may prove particularly useful for the clinical assessment of CSF1R inhibitor pharmacodynamics within target tissues.

Efficacy of JNJ-40346527 in TCT colitis was consistent with previous investigations of the role of CSF-1/CSF-1R in a second IBD model. Specifically, CSF-1-deficient mice and male CSF-1R^+/-^ mice were reported to be less susceptible to dextran sulfate sodium (DSS)-induced colitis,[17, 18] and treatment with a CSF-1 neutralizing antibody or a CSF-1R blocking antibody was highly efficacious in this same model.[19]

Macrophage lineage cells in IBD and in healthy intestinal mucosa have been intensively investigated, [6, 7, 9, 31, 32] and results indicate the gastrointestinal tract may be unique in terms of macrophage ontogeny. In most tissues, resident macrophages retain proliferative capacity throughout adult life and are maintained by self-renewal *in situ* with little contribution from the circulating monocytes.[33–36] However, using fate mapping in mice, Bain et al. reported that colonic resident macrophages were non-proliferative and were replenished from CCR2^+^ Ly6C^hi^ bone marrow-derived monocytes.[11] Shortly after extravasation, infiltrating monocytes transiently expressed markers consistent with a pro-inflammatory phenotype,[5] but, over the next 76–96 hr, differentiated through a series of intermediaries into mature macrophages, acquiring MHCII expression, down-regulating Ly6C, and upregulating CD64, F4/80 and CX3CR1 expression. These F4/80^hi^ CX3CR1^hi^ cells produced IL-10 and adopted the anti-inflammatory phenotype associated with resident macrophages. However; during colitis, macrophage numbers in the colon increased several fold.[5] The increase was attributed to cells derived from the same infiltrating CCR2^+^ Ly6C^hi^ monocytes that replenish resident macrophages in the healthy intestine. These newly recruited cells, described as F4/80^int^ CX3CR1^int^, become the dominant subset and support key pathogenic mechanisms in colitis through robust expression of TNFα and IL-23 and efficient activation of T cells through MHCII. In the context of an inflamed tissue micro-environment, this population fails to transition efficiently to F4/80^hi^ CX3CR1^hi^ homeostatic cells, [5, 8, 32] and these cells persist as a source of “586” inflammatory signaling. An analogous population with high expression of CD14 has been described in human CD.[10]

M1 and M0 macrophages express F4/80 antigen, as do monocytes, and IHC of TCT colitis mucosa indicated a deep reduction in F4/80^+^ cells in mice treated with JNJ-40346527. The conclusion that M1 and M0 macrophages were reduced was supported by sharp reductions in GSVA enrichment scores for gene sets indicative of these populations. Meanwhile, the strong reduction in CCR2 and Ly6c mRNA expression in the mucosa suggests attenuated recruitment of monocytes. CSF-1 is known to induce expression of CCL2 and CCL7[37]. These are the two ligands mediating monocyte recruitment through CCR2, and reduced expression of CCL2 and CCL7 mRNA in JNJ-40346527-treated mice provide one potential mechanism for reduced monocyte/M1 macrophage recruitment. Overall, the ability of JNJ-40346527 to depress inflammatory M1 macrophages is consistent with previous reports that anti-CSF1R blocking antibodies block inflammatory macrophage recruitment in models of skin, lung, and peritoneal inflammation,[38–40] although not all studies agree.[41]

The majority (~90%) of blood monocytes are Ly6C^hi^ in mice and CD14^+^ CD16^-^ in humans. Bone marrow production of these “classical” monocytes is not CSF-1R-dependent, and although they are precursors of inflammatory macrophages in colitis, they are also precursors of a minor (~10%) population of “nonclassical” monocytes that are Ly6C^lo^ in mice and CD14^lo^ CD16^+^ in humans and that have increased potential to express TNFα. The maturation step yielding nonclassical blood monocytes is CSF-1R-dependent and inhibited in mice and in humans by anti-CSF-1R antibodies and by JNJ-40346527.[21, 41] A subset of inflammatory CD14^+^ macrophages in CD mucosa are CD16^+^.[42] Whether recruited from nonclassical monocytes, or differentiated in situ, the relationship of these cells to nonclassical monocytes and their dependence on CSF-1R merits further study. Other functionally complex immature myeloid subsets in blood and inflamed tissues include neutrophilic and monocytic myeloid-derived suppressor cells (MDSC). Murine studies associate CSF-1R inhibition with increased recruitment of neutrophilic MDSC to tumors.[43] MDSC may be pro or anti-inflammatory depending on context, and their role in IBD is poorly understood. The contribution of MDSC to the action of JNJ-40346527 in TCT colitis requires further study, although it is clear overall neutrophil recruitment was reduced.

Whilst resident macrophages are considered protective in healthy mucosa, their function during ongoing inflammation has received less attention. Mature macrophages exhibit a high degree of phenotypic plasticity.[44] This was highlighted in studies exploring acute models of intestinal inflammation, where increased epithelial permeability accompanied by the uptake of bacterial antigen by resident macrophages, led to macrophage activation and a shift to the production of pro-inflammatory cytokines.[45, 46] The degree to which this might occur in IBD has not been specifically addressed, but the ability to induce reprogramming of the pro-inflammatory and anti-inflammatory phenotypes in response to the changing tissue microenvironment has been reported *in vitro*[47] and reviewed.[48] Thus, it is possible that resident macrophages may contribute to disease and depletion of these cells may account for part of the anti-inflammatory activity of JNJ-40346527.

IPA analysis of the TCT colitis and the CD disease signatures revealed striking concordance at the pathway level. In CD, anti-TNFα therapy reversed the disease-associated pathways in responding patients, and the effect of JNJ-40346527 in mice was similar to that achieved by anti-TNFα in humans. Together, the analyses indicate CSF-1R inhibition may be effective in treating CD. Application of the CSF-1 and TNFα gene sets to the TCT colitis expression data revealed both differences as well as similarities in the mechanistic impact of JNJ-40346527 and anti-TNFα. GSVA indicated that the CSF-1 gene set was enriched in TCT colitis, only weakly and non-significantly impacted by anti-TNFα therapy, but robustly normalized following treatment with JNJ-40346527. At the same time, suppression of the M1 and M0 gene sets by JNJ-40346527 and by anti-TNFα indicate their mechanisms of action may intersect by addressing inflammatory macrophages, albeit through different biological mechanisms. Infliximab and CNTO5048 bind to both soluble and transmembrane TNFα to prevent interaction of TNFα with its receptor and the induction of proinflammatory signaling.[49] In addition, binding of infliximab to transmembrane TNFα upregulated on activated macrophages and lymphocytes can trigger antibody‐dependent cellular toxicity or complement‐dependent cytotoxicity mechanisms leading to apoptosis. Thus, TNFα antibody therapy may partly eliminate proinflammatory macrophages along with other components of the immune cell repertoire. This is supported by GSVA analyses in CD responders where the M1 gene signature was reduced to baseline by infliximab. While the mechanisms underlying anti-TNFα non-response are not clear, patients who failed to respond to infliximab had exaggerated CSF-1 and M1 gene signatures prior to treatment, and treatment had no effect (Fig 1 and S2 Fig). The data suggest that inflammatory macrophages in this population cannot be controlled by anti-TNFα alone and control may require a second complementary mechanism such as CSF-1R inhibition. These observations indicate that multiple mechanisms drive disease pathology in IBD, and that enhanced CSF-1/CSF-1R signaling could represent a differential disease mechanism within select IBD patients.

Taken together, our findings suggest that inhibition of CSF-1/CSF-1R signaling by JNJ-40346527 may be effective in managing inflammatory bowel disease. CSF-1 and TNFα may mediate pathogenic processes that exhibit different degrees of dominance in some patient subsets, and this compound may be of particular benefit for patients who fail anti-TNFα therapy. To further investigate the hypothesis, JNJ-40346527 (PRV-6527) is currently being tested in a randomized, double-blind, placebo-controlled Phase 2 clinical trial in Crohn’s disease (EudraCT number: 2017-003017-25).

## Supporting information

S1 Text Method for determining the histopathology sum of scores and neutrophil infiltrate fraction. (DOCX)

S1 Table Description of experimental groups in TCT colitis studies. (DOCX)

S2 Table Differential expression of M1 macrophage gene set genes in TCT colitis. (DOCX)

S3 Table Differential expression of M0 macrophage gene set genes in TCT colitis. (DOCX)

S4 Table Differential expression of Th1 lymphocyte gene set genes in TCT colitis. (DOCX)

S5 Table Differential expression of CSF-1 gene set genes in CD and TCT colitis. (DOCX)

S6 Table Individual component histopathology scores used to calculate the sum of scores for TCT colitis studies. (DOCX)

S7 Table Differential gene expression statistics from RNAseq of TCT colitis study #2. (DOCX)

S8 Table Z scores for IPA pathway analysis of CD and T cell reconstituted colitic mouse datasets. (XLSX)

S1 Fig Combined results from two additional TCT colitis studies showing weight loss in the PBS vehicle treated group. (TIFF)

S2 Fig GSVA of M1 and M0 gene sets in CD mucosa. (TIFF)
