# Supplementary material for: The CSF-1-receptor inhibitor, JNJ-40346527 (PRV-6527), reduced inflammatory macrophage recruitment to the intestinal mucosa and suppressed murine T cell mediated colitis
Source: PLoS One. 2019 Nov 11;14(11):e0223918. doi: 10.1371/journal.pone.0223918 (PMC6844469; doi:10.1371/journal.pone.0223918)
Supplement: S1 Text — (DOCX) [file pone.0223918.s001.docx]

S1 Text

Histopathology was performed on colon tissues from groups 1–7. For each H&E stained section,

submucosal edema was quantitated by measuring the distance from the muscularis mucosa to the

internal border of the outer muscle layer in a non-tangential area thought to most representative

the severity of this change. Mucosal thickness was also measured in a non-tangential area of the

section that best represented the overall mucosal thickness. This parameter is indicative of gland

elongation and mucosal hyperplasia. To incorporate this parameter into the summed score, a hyperplasia score was derived from the measurement as follows:

0 = <200 μm

1 = 200–299 μm

2 = 300–449 μm

3 = 450–599 μm

4 = 600–699 μm

5 = ≥700 μm

The extent of inflammation [macrophage, lymphocyte and polymorphonuclear leukocyte cell

(PMN) infiltrate] was assigned severity scores according to the following criteria:

0 = Normal

0.5 = Very Minimal, 1 or 2 small foci, mononuclear inflammatory cells (MNIC)

likely background mucosal lymphoid aggregates

1 = Minimal, larger focal area with MNIC and neutrophils or minimal diffuse, no

separation of glands, may be mostly in areas of submucosal edema or mesentery

2 = Mild, diffuse mild, or multifocal affecting 11–25% of mucosa with minor focal

or multifocal gland separation, no separation in most areas

3 = Moderate, 26–50% of mucosa affected with minimal to mild focal or multifocal

separation of glands by inflammatory cell infiltrate, milder in remaining areas

of mucosa with some areas having no gland separation by inflammation

4 = Marked, 51–75% of mucosa affected with mild to moderate separation of

glands by inflammatory cell infiltrate, minimal to mild in remaining areas of

mucosa but all glands have some separation by infiltrate

5 = Severe, 76–100% of mucosa affected with moderate to marked areas of gland

separation by inflammatory cell infiltrate, mild to moderate in remaining areas

of mucosa

The number of definite mucosal lymphoid aggregates (GALT, Peyer’s patches) were

quantified to determine any group differences. In general, normal/disease free small

intestine sections have 0–1 aggregate; however, for comparison purposes, all “normal” section

small aggregates (generally 1) are labeled as abnormal and given inflammation scores of 0.5 to

document the presence of these aggregates along with the count of aggregates.

The parameters reflecting epithelial cell loss/damage (gland loss or erosion) were scored

individually using a percent area involved scoring method:

0 = None

0.5 = Very Minimal, 1 or 2 small focal areas of gland loss or mucosal erosion

1 = Minimal, 1–10% of the mucosa affected

2 = Mild, 11–25% of the mucosa affected

3 = Moderate, 26–50% of the mucosa affected

4 = Marked, 51–75% of the mucosa affected

5 = Severe, 76–100% of the mucosa affected

Colon glandular loss - this includes crypt epithelial as well as remaining gland epithelial loss.

Colon Erosion - this reflects loss of surface epithelium and generally is associated with mucosal

hemorrhage (reflective of the bleeding seen clinically and at necropsy).

The 4 important scored parameters (inflammation, glandular loss, erosion, hyperplasia) were

ultimately summed to arrive at a sum of histopathology scores, which indicates the overall

damage and has a maximum score of 20.

Inflammatory cell infiltrates in the colonic mucosa were evaluated for approximate percent of

neutrophils in the total infiltrate by examining 3 to 4 representative fields of view per tissue section using a grid reticle. PMN were differentiated from mononuclear cells by nuclear morphology. The percentage of the grid filled by PMN compared to total infiltrate was estimated and results were averaged for each section.
