# Supplementary material for: The CSF-1-receptor inhibitor, JNJ-40346527 (PRV-6527), reduced inflammatory macrophage recruitment to the intestinal mucosa and suppressed murine T cell mediated colitis
Source: PLoS One. 2019 Nov 11;14(11):e0223918. doi: 10.1371/journal.pone.0223918 (PMC6844469; doi:10.1371/journal.pone.0223918)
Supplement: S1 Table — (DOCX) [file pone.0223918.s002.docx]

S1 Table

| **Study #1** | | | | |
| --- | --- | --- | --- | --- |
| **Strain** | **TCT** | **Treatment** | N | **Endpoints** |
| C.B-17/SCID | no | None | 4 | BW, Colon W/L, histopath |
| C.B-17/SCID | yes | vehicle QID d14-42 PO | 10 | BW, Colon W/L, histopath |
| C.B-17/SCID | yes | JNJ527, 5 mg/kg QID d14-42 PO | 10 | BW, Colon W/L, histopath |
| C.B-17/SCID | yes | JNJ527, 10 mg/kg QID d14-42 PO | 10 | BW, Colon W/L, histopath |
| C.B-17/SCID | yes | JNJ527, 20 mg/kg QID d14-42 PO | 10 | BW, Colon W/L, histopath |
| **Study #2** | | | | |
| **Strain** | **TCT** | **Treatment** | N | **Endpoints** |
| C.B-17/SCID | no | None | 8 | BW, Colon W/L, histopath, IHC |
| C.B-17/SCID | yes | vehicle QID d14-42 PO | 10 | BW, Colon W/L, histopath, IHC |
| C.B-17/SCID | yes | JNJ527, 15 mg/kg QID d14-42 PO | 10 | BW, Colon W/L, histopath, IHC |
| C.B-17/SCID | yes | Vehicle QID d14-20, JNJ527, 15 mg/kg QID d21-42 PO | 10 | BW, Colon W/L, histopath, IHC |
| C.B-17/SCID | yes | PBS Q3D, d21-42 | 8 | BW, Colon W/L, histopath |
| C.B-17/SCID | yes | Isotype control, 3 mg/kg Q3D, d21-42 | 10 | BW, Colon W/L, histopath |
| C.B-17/SCID | yes | CNTO5048, 3 mg/kg Q3D, d21-42 | 10 | BW, Colon W/L, histopath |
| Balb/c | no | None | 6 | RNAseq |
| C.B-17/SCID | no | None | 6 | RNAseq |
| C.B-17/SCID | yes | vehicle QID, d14-42 PO | 8 | RNAseq |
| C.B-17/SCID | yes | JNJ527, 15 mg/kg QID, d21-42 PO | 8 | RNAseq |
| C.B-17/SCID | yes | Isotype control, 3 mg/kg Q3D,  d21-42 IP | 6 | RNAseq |
| C.B-17/SCID | yes | CNTO5048, 3 mg/kg Q3D, d21-42 IP | 8 | RNAseq |
