# Supplementary material for: The CSF-1-receptor inhibitor, JNJ-40346527 (PRV-6527), reduced inflammatory macrophage recruitment to the intestinal mucosa and suppressed murine T cell mediated colitis
Source: PLoS One. 2019 Nov 11;14(11):e0223918. doi: 10.1371/journal.pone.0223918 (PMC6844469; doi:10.1371/journal.pone.0223918)
Supplement: S6 Table — (DOCX) [file pone.0223918.s007.docx]

| S6 Table | | | | | | |
| --- | --- | --- | --- | --- | --- | --- |
| Study #1 |  | n | Inflammation | Glandular loss | Erosion | Hyperplasia |
| SCID/no TCT | no treatment | 4 | 0.0 | 0.0 | 0.0 | 0.0 |
| SCID/TCT | Vehicle | 10 | 3.8 | 1.8 | 0.1 | 2.4 |
| SCID/TCT | JNJ527, 5 mg/kg | 10 | 2.3 * | 1.0 | 0.1 | 1.2 * |
| SCID/TCT | JNJ527, 10 mg/kg | 10 | 2.1 * | 0.7 * | 0.1 | 1.4 * |
| SCID/TCT | JNJ527, 20 mg/kg | 10 | 1.9 * | 0.4 * | 0.0 | 1.3 * |
| Study #2 |  | n | Inflammation | Glandular loss | Erosion | Hyperplasia |
| SCID/no TCT | no treatment | 8 | 0 | 0 | 0 | 0 |
| SCID/TCT | Vehicle | 10 | 2.9 | 2.0 | 0.1 | 1.0 |
| SCID/TCT | JNJ527, d21-42 | 10 | 1.2 * | 0.5 * | 0 | 0.2 * |
| SCID/TCT | JNJ527, d14-42 | 10 | 1.4 * | 0.7 * | 0.0 | 0.4 |
| SCID/TCT | PBS | 8 | 2.7 | 1.8 | 0.0 | 0.6 |
| SCID/TCT | Isotype control | 10 | 3.4 | 2.4 | 0.1 | 1.3 |
| SCID/TCT | CNTO5048 | 10 | 0.5 * | 0.1 * | 0.0 | 0.0 |
| * p<0.5 vs. Vehicle; TCT = T cell transfer (CD45^high^ T cells) | | | | | | |
