# Supplementary material for: The CSF-1-receptor inhibitor, JNJ-40346527 (PRV-6527), reduced inflammatory macrophage recruitment to the intestinal mucosa and suppressed murine T cell mediated colitis
Source: PLoS One. 2019 Nov 11;14(11):e0223918. doi: 10.1371/journal.pone.0223918 (PMC6844469; doi:10.1371/journal.pone.0223918)
Supplement: S7 Table — (DOCX) [file pone.0223918.s008.docx]

| S7 Table | | | |
| --- | --- | --- | --- |
| Comparison | Up ≥ 2-fold  FDR ≤ 0.05 | Down ≥ 2-fold  FDR ≤ 0.05 | Total ≥ 2-fold  FDR ≤ 0.05 |
| SCID TCT_Vehicle vs. SCID_naive | 1377 | 883 | 2260 |
| SCID TCT_JNJ527 vs. SCID_naive | 58 | 75 | 133 |
| SCID TCT_Isotype control vs. SCID_naive | 1079 | 254 | 1333 |
| SCID TCT_CNTO5048 vs. SCID_naive | 53 | 11 | 64 |
| SCID TCT_JNJ527 vs. SCID TCT_Vehicle | 391 | 700 | 1091 |
| SCID TCT_CNTO5048 vs. SCID TCT_Vehicle | 492 | 788 | 1280 |
| SCID_naive vs Balb/c | 7 | 36 | 43 |
