# Supplementary figures and images for: The CSF-1-receptor inhibitor, JNJ-40346527 (PRV-6527), reduced inflammatory macrophage recruitment to the intestinal mucosa and suppressed murine T cell mediated colitis
Source: PLoS One. 2019 Nov 11;14(11):e0223918. doi: 10.1371/journal.pone.0223918 (PMC6844469; doi:10.1371/journal.pone.0223918)

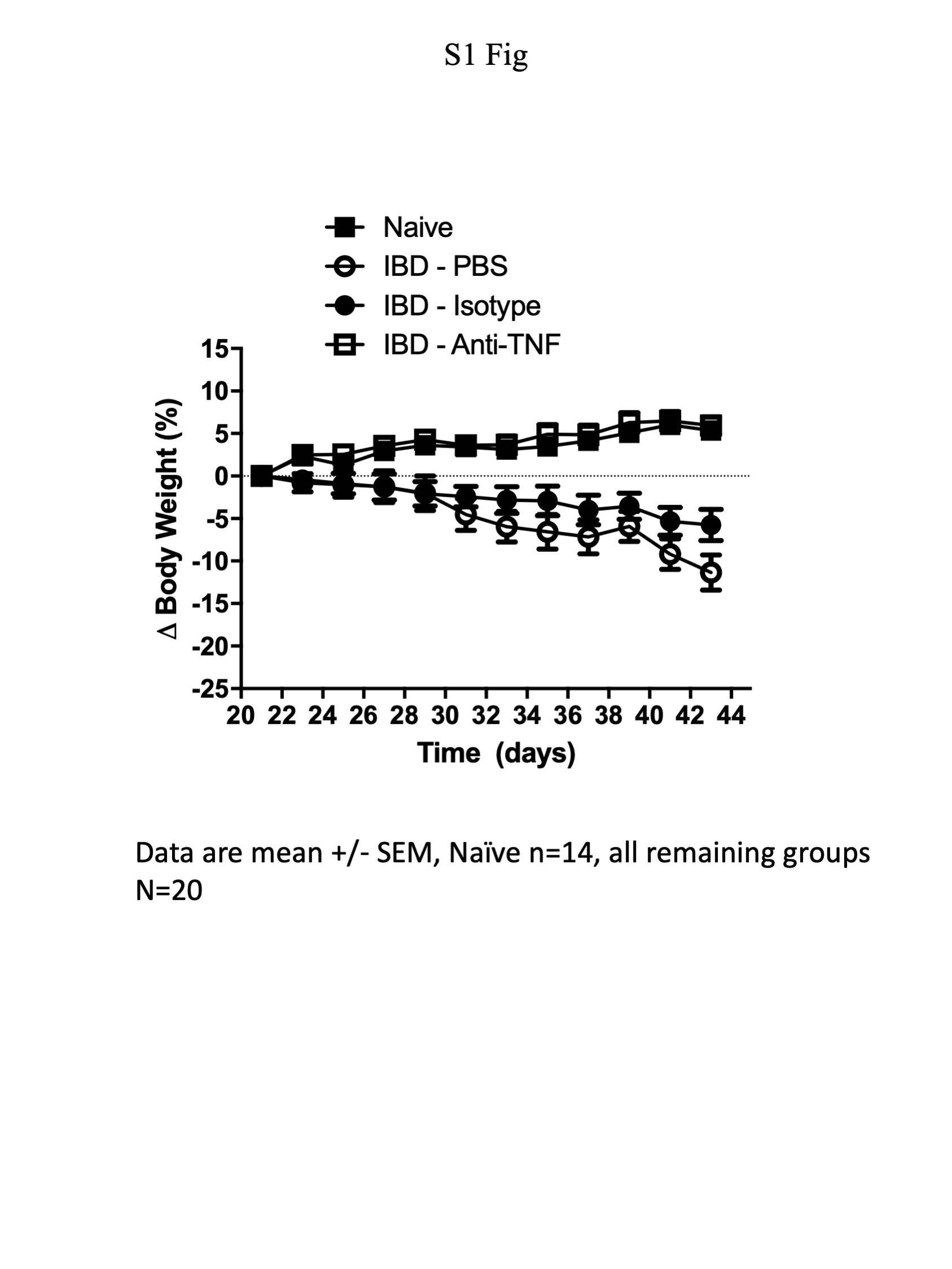

Supplement: S1 Fig — (TIFF) [file pone.0223918.s010.tiff]

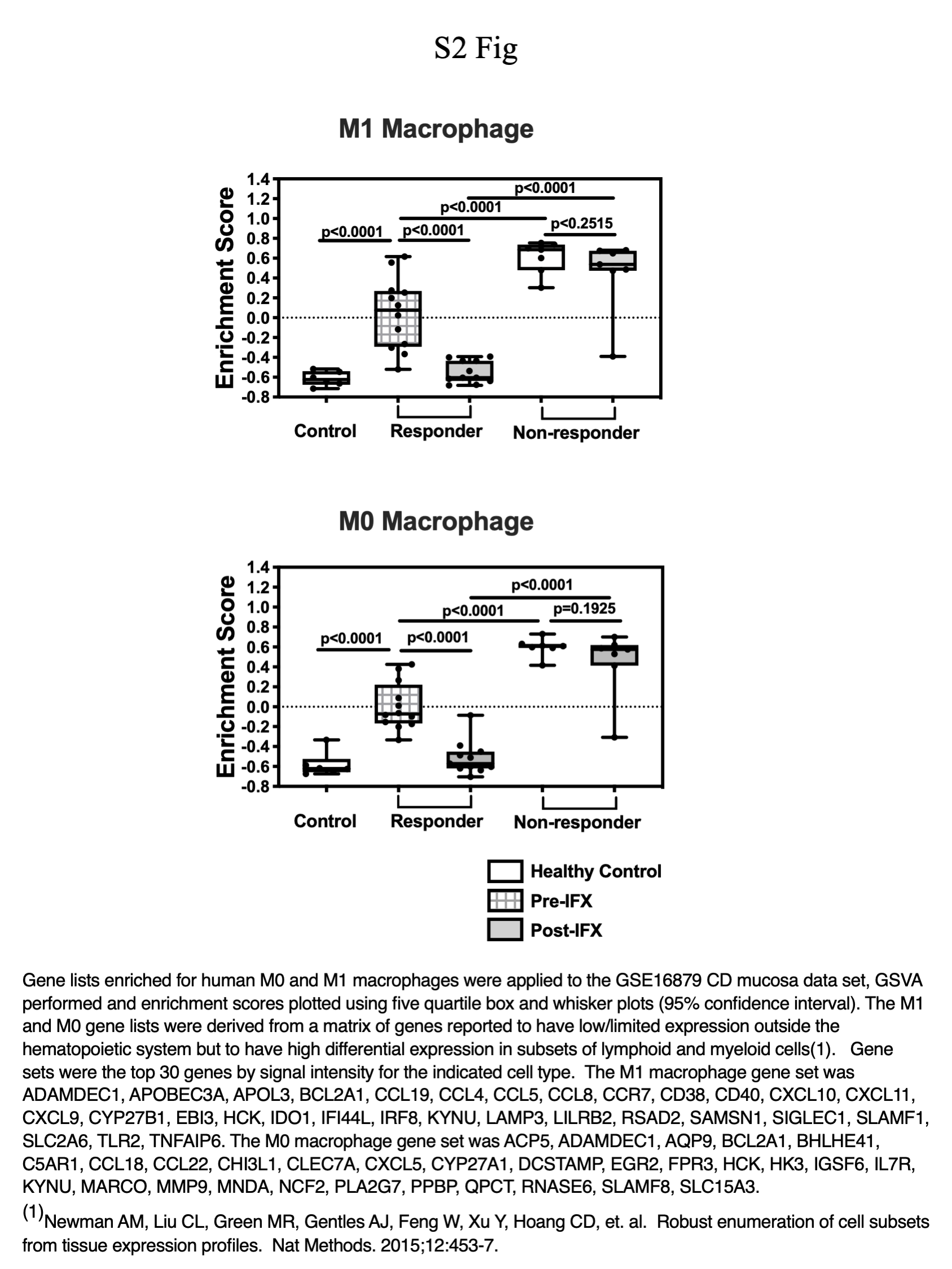

Supplement: S2 Fig — (TIFF) [file pone.0223918.s011.tiff]
